# Adapting Lymphedema Treatment in Patients with a Mental Disability

**DOI:** 10.1155/2017/4958127

**Published:** 2017-09-11

**Authors:** Maria de Fátima Guerreiro Godoy, Lívia Maria Pereira de Godoy, Rodrigo Ocampos Troitino, José Maria Pereira de Godoy

**Affiliations:** ^1^Medicine School in São José do Rio Preto (FAMERP), São José do Rio Preto, SP, Brazil; ^2^Research Group of Clínica Godoy, São José do Rio Preto, SP, Brazil; ^3^Medicine School of Marilia (FAMEMA), Marilia, SP, Brazil; ^4^Medicine School of ABC, Santo Andre, SP, Brazil; ^5^Cardiology and Cardiovascular Surgery Department, Medicine School in São José do Rio Preto (FAMERP), São José do Rio Preto, SP, Brazil

## Abstract

**Background and Purpose:**

Mental disability is often characterized by significant limitations in adaptive skills. When this condition is associated with lymphedema, treatment requires greater commitment of the care team. The objective of this study is to report the treatment of lymphedema using only one therapeutic technique, a low-stretch grosgrain stocking.

**Case Report:**

We report the case of a 14-year-old mentally challenged female patient with lymphedema of the left leg, motor difficulties, and impaired speech and sight. According to the caregiver, lymphedema was present at birth; however, the patient had not been submitted to specific treatment. Thus, only one technique, an adapted low-stretch grosgrain compression stocking, was proposed as it could be used during daily life activities. The adaptation involved the grosgrain stocking, fastened using eyelets and cord up to the thigh, being sewn onto a pair of cotton shorts. The result was a clinical improvement with reductions in the perimeter and volume due to the compliance of the patient and the family to treatment.

**Conclusion:**

The use of a single treatment strategy in the form of a low-stretch stocking in such cases together with the involvement of a multidisciplinary team can lead to good treatment outcomes for chronic lymphedema.

## 1. Introduction

Mental disability is characterized by lower than average intellectual functioning, intelligence quotient (IQ) less than 70, the presence of significant limitations in two or more areas of adaptive skills, and its onset before the age of 18 years [1, 2]. Its aetiology includes any condition that impairs brain development, whether genetic, due to problems during pregnancy, problems at birth or after, and socioeconomic conditions such as malnutrition and lack of stimulation [3].

The main symptoms of the individual with mental disability are related to their impaired adaptive functioning, that is, how patients deal with the common demands of life and the degree to which they meet the criteria of personal independence expected of someone in their age group and sociocultural condition in the community context. These aspects are influenced in part by the degree of education, motivation, personality, and social and professional opportunities [1].

Lymphedema is characterized by the abnormal accumulation of protein-rich fluid in the tissues resulting from dysfunction of the lymphatic system that leads to an imbalance between the formation of lymph and its absorption in the initial lymphatics [4].

The most used classification for lymphedema is divided into four stages. Stage 0 is a latent or subclinical phase. In Stage I, the patient wakes up without oedema but swelling develops over the day. In Stage II, the patient wakes up with the leg swollen with the swelling getting worse during the day. Finally, Stage III (elephantiasis) is a more advanced stage than Stage II in which deformities appear. Classification by severity can also be considered where the volume of the oedematous limb is compared to the contralateral limb: mild < 20%; moderate (20–40%); and severe > 40% [5].

An association of therapies is recommended in the treatment of lymphedema, including manual and mechanical lymph drainage, myolymphokinetic exercises, compression therapy, and hygienic, nutritional, and psychological care [6]. However, in mentally challenged patients, treatment requires greater commitment from the care team and it is often necessary to adapt therapy to the difficulties of the disability. Thus, because of the evolutionary nature of the disease, much care and total commitment to therapy are required, making the guidance and support of a multiprofessional team essential for patient adhesion and therapeutic success. The objective of this study is to report on the reduction of lymphedema using just one form of therapy, a low-stretch grosgrain stocking adapted for this patient, and support of a multiprofessional team.

## 2. Case Report

**Figure 1:**
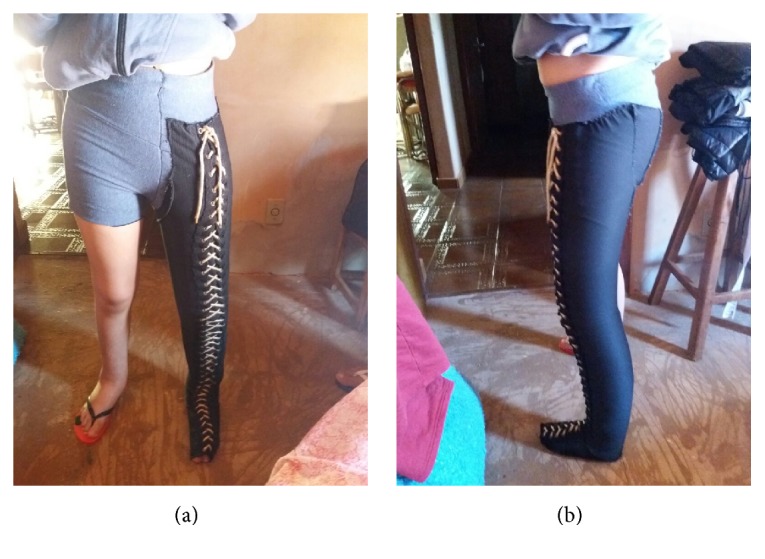
Adaptation of the grosgrain stocking with cotton shorts (front and side view) that made daily use possible.

We report on the evolution of lymphedema treatment in a patient with special needs using with just one form of therapy, a low-stretch grosgrain stocking. Grosgrain is a cotton-polyester ribbed textile that has low elasticity (<50) across the material but is elastic along the material and thus it allows low-stretch compression [7]. The homemade stockings used in this study constantly require readjustment because of reductions in the size of the limb (Figure 1). This 14-year-old female patient had stage III lymphedema including fibroses (elephantiasis) of the left lower limb, mental disability, motor difficulties, and impaired speech and sight. According to the caregiver, the lymphedema was present at birth; however, the patient had not been submitted to any specific treatment. Intensive treatment with an association of techniques was not possible due to the motor and behavioural limitations. Thus, a single therapy was proposed with the adaptation of compression therapy using a specific fabric called grosgrain that could be used during the patient's daily activities, both at home and at her school. It was necessary to counsel the entire family, particularly the mother and caregiver, about skin care using a neutral moisturizer cream without chemicals before donning the stocking and training to uniformly adjust the fastening with eyelets and cord. The caregivers were advised about the importance of removing the stocking twice a day to wash the leg and check the skin in order to improve patient compliance. The involvement of a transdisciplinary team to give guidance and support to the caregiver was fundamental. The result was clinical improvement with perimetric and volumetric reductions. Evaluations were performed by volumetry using the water displacement technique and measurement of the limb circumference weekly in the first month (Table 1), then every two weeks, and eventually every three months after normalization of the limb volume because the patient and the family complied well to treatment. This study was approved by the Research Ethics Committee of the Medical School in São José do Rio Preto number 007016/2017.

## 3. Discussion

The present study reports the therapeutic evolution of lower limb lymphedema of a teenager with a severe mental disability and physical limitations aggravated by the presence of lymphedema. Adaptation of the therapy was essential for the success of the treatment because of the limitations of the patient, which restricted the use of the techniques generally employed to treat lymphedema. The involvement of the family was essential for the patient to accept the stocking made of grosgrain fabric.

In relation to physical activities, the patient's special school was advised as to the necessity of stimulating the patient to perform physical activities while wearing the grosgrain stocking. Part of the treatment was developed in this way. Over time, she began to accept and create a bond with the care team and started performing mechanical lymphatic therapy, but she did not allow manual lymph drainage or cervical stimulation. She felt uncomfortable with anyone touching her body and began to show intolerant behaviour by screaming. Thus, mechanical lymphatic therapy using an apparatus that performs passive ankle flexion and extension movements [8] was important; when she accepted this treatment, she started to use it at her home every day. However, she was always accompanied by her mother or relatives who she trusted. There are almost no reports in the literature describing the treatment of lymphedema in adolescents with this type of problem, but treatment of these patients is important.

The treatment of lymphedema in a mentally challenged patient has been shown to be effective when the objectives of the underlying disease are achieved, such as those related to rejection and stigmatization of the patient with consequent integration into society. The use and development of this individual's potential with the help of professional support and the possibility that the patient feels complete and integrated in society maximize adherence to lymphedema treatment and, consequently, provide results that are often better than expected.

Incomprehension of the possible capacities of mentally challenged people and the special care that they need, as well as the inconsistency and insufficiency of community policies to meet their demands, hinder the social integration of these patients and, consequently, their physical treatment and their psychosocial evolution.

The feeling of frustration and distress and the reactions of denial, anger, sadness, guilt, and anxiety of the relatives of mentally disabled individuals associated with a progressive deformity of a lymphedematous limb interfere with their affective attachment to these individuals. In addition, there is a financial burden, loss of professional opportunities, and even social isolation of the family because of this patient [9, 10]. However, one cannot block family involvement. It is extremely important that the family member responsible for the patient receives guidance and continuously reminds the patient about the care necessary to treat lymphedema.

## 4. Conclusion

The use of a single treatment strategy in the form of a low-stretch stocking in cases such as this mentally and physically challenged child together with the involvement of a multidisciplinary team can lead to good treatment outcomes for chronic lymphedema.

## Figures and Tables

**Table 1:** Initial volume and weekly reductions in volume with treatment.

	Volume (mL)	Volume (mL)	Difference (mL)
	Right leg	Left leg	
Initial	2705	4478	1773
1 week	2573	3770	1197
2 weeks	2659	3215	556
3 weeks	2670	2991	321
4 weeks	2693	2962	269
